# Supplementary material for: Deciphering Transcriptional Networks during Human Cardiac Development
Source: Cells. 2022 Dec 3;11(23):3915. doi: 10.3390/cells11233915 (PMC9739390; doi:10.3390/cells11233915)
Supplement: Supplementary file 1 [file cells-11-03915-s001.zip › Supplementary Figures.pdf]

Supplemental information

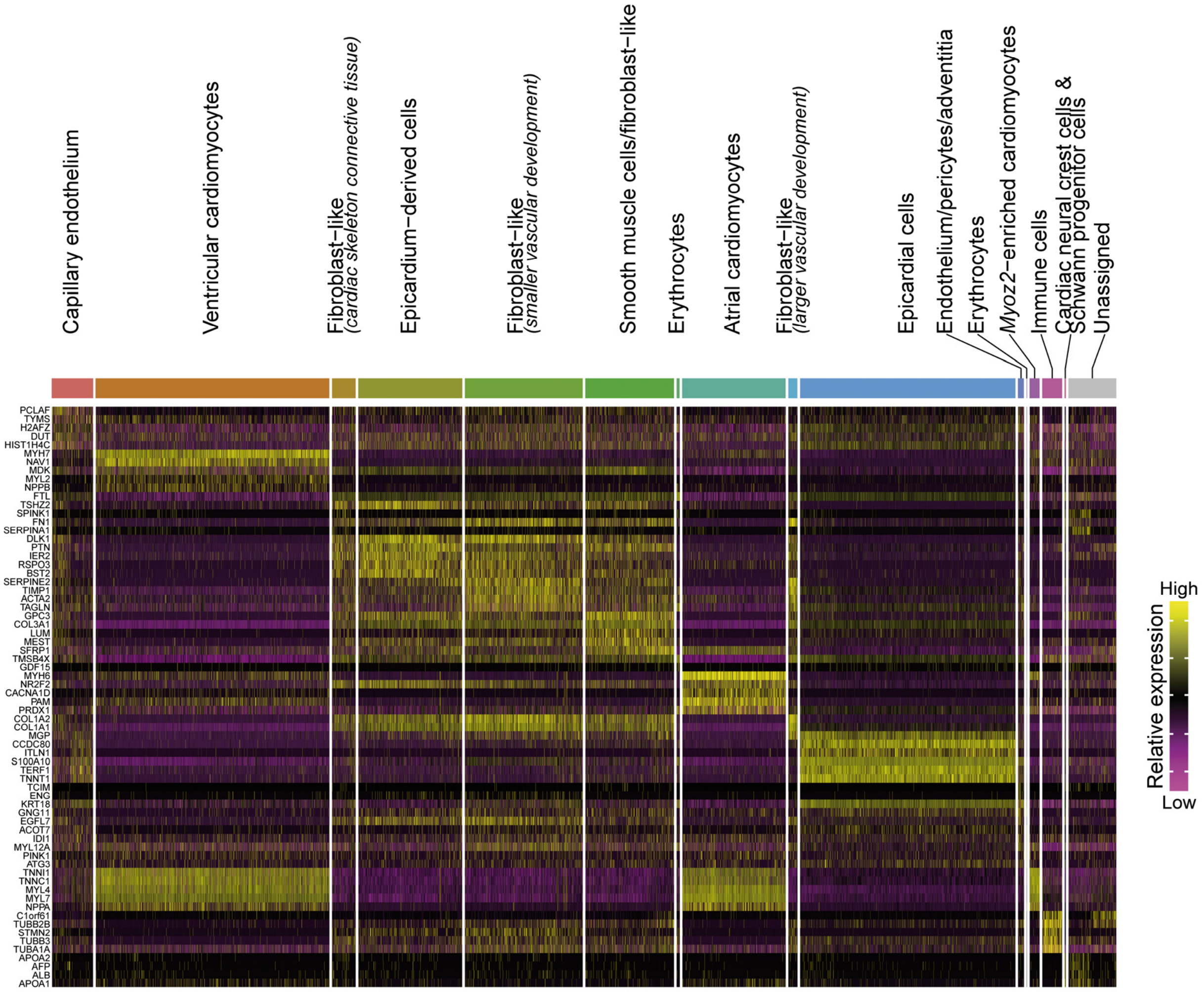

Figure S1 – Transcriptomic signatures of cell types generated by hiPSC cardiac differentiation.

Heatmap displaying expression levels of the top 5 markers of each cell population found in Fig 1D. Markers were identified using the FindAllMarkers function from the R package Seurat.

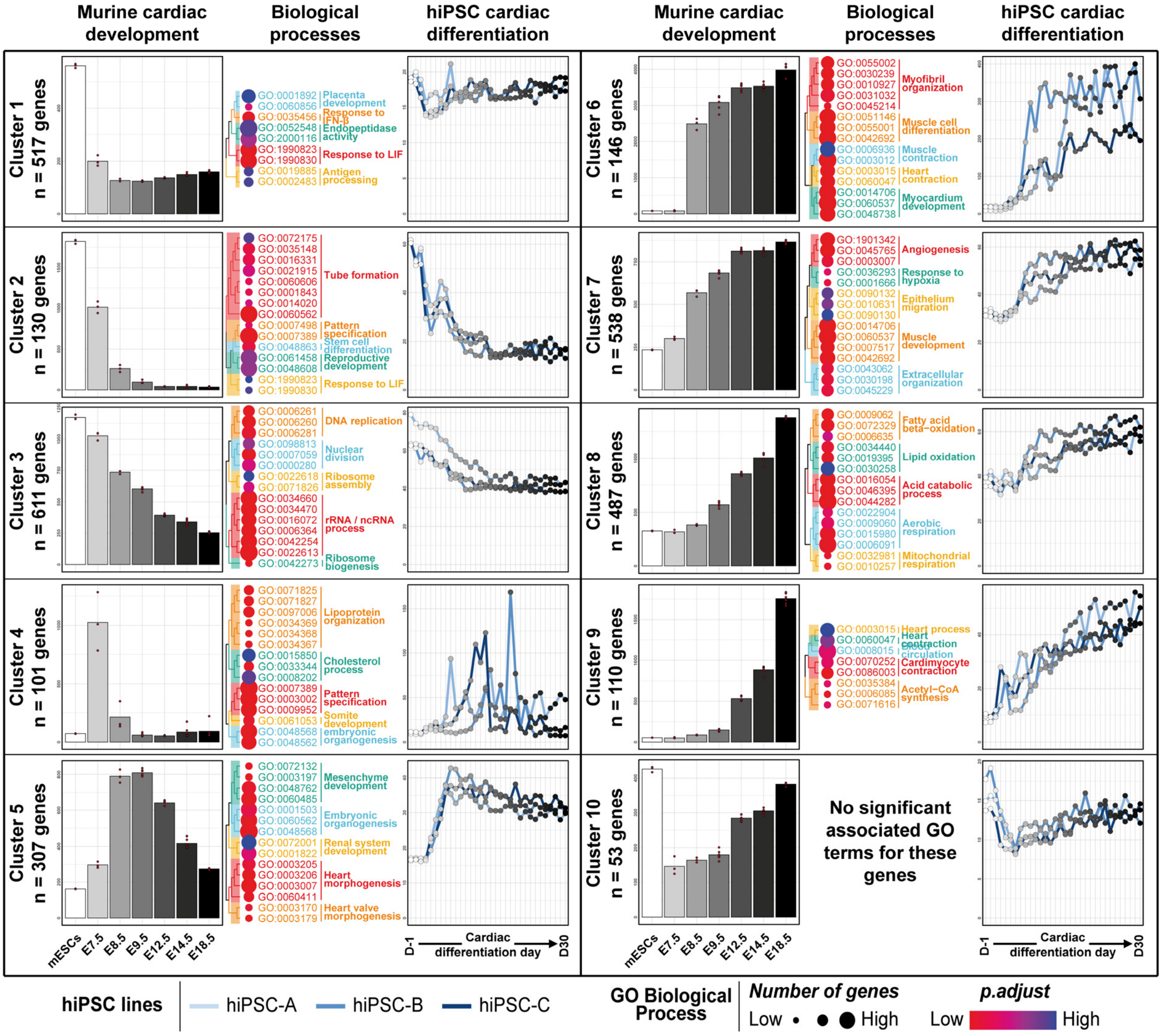

Figure S2 – Expression profile and functional annotation of the top 3 000 differentially expressed genes during murine cardiac development, and comparison with hiPSC cardiac differentiation gene expression dataset.

For each cluster, average gene expression level during murine cardiac development (left panel for each cluster) and their human orthologs during hiPSC cardiac differentiation (right panel for each cluster) are shown. Replicates of gene expression levels were averaged for murine data (n=3 to n=6 per timepoint, depending on the stage) and hiPSC cardiac differentiation (n=3 per hiPSC line and per timepoint). The 15 most significantly related GO terms are displayed on the middle panel for each cluster.

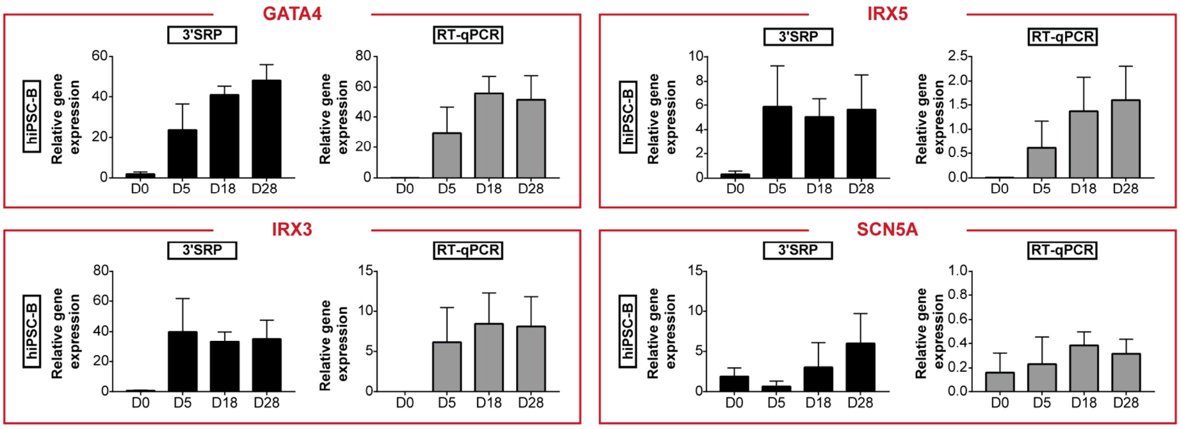

Figure S3 – RT-qPCR confirmation of bulk transcriptomic data.

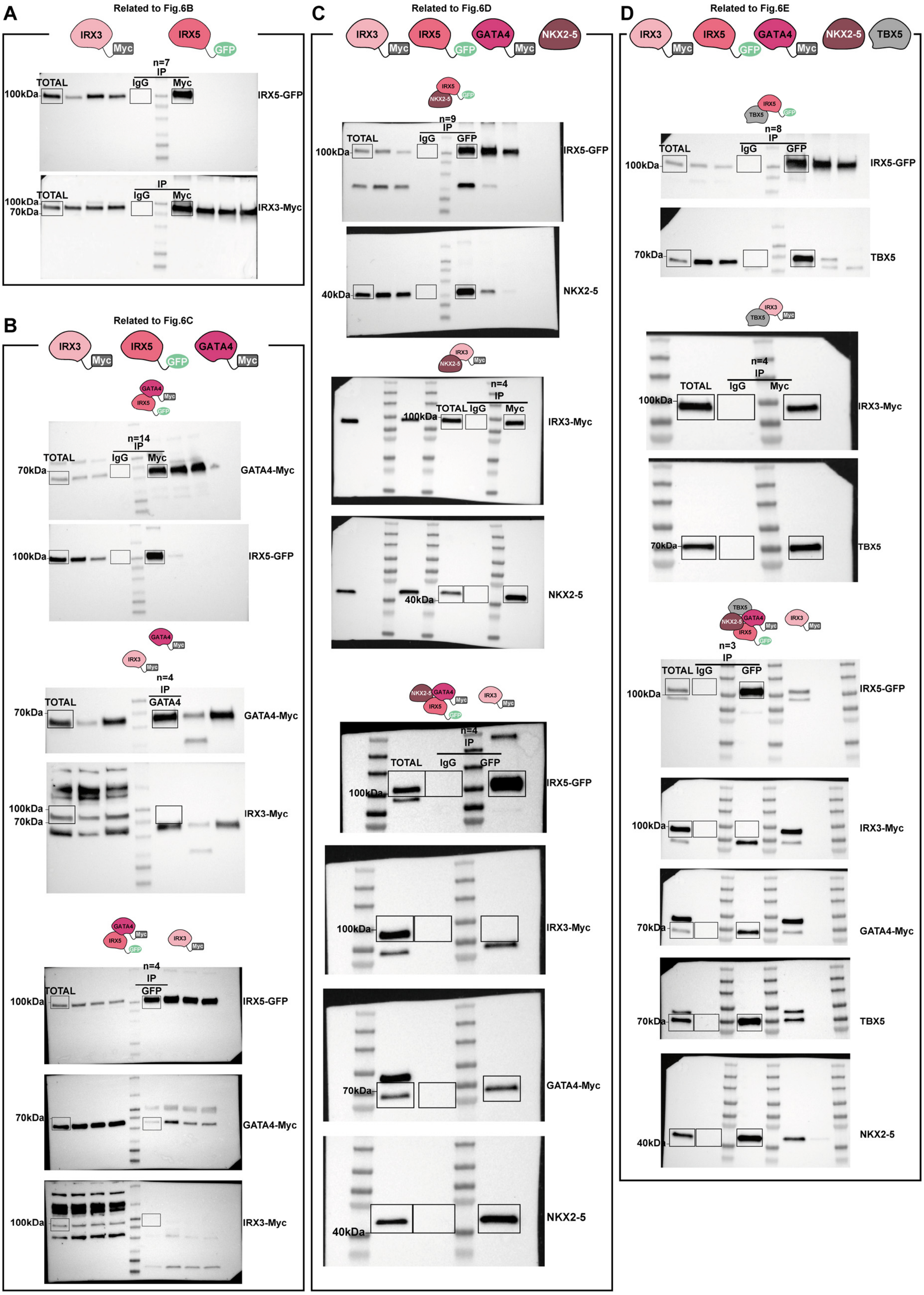

Figure S4 – Original western blots related to Figure 6.
